# Post-traumatic stress disorder among healthcare workers during the COVID-19 pandemic in Italy: Effectiveness of an eye movement desensitization and reprocessing intervention protocol

**DOI:** 10.3389/fpsyg.2022.964334

**Published:** 2022-09-08

**Authors:** Isabel Fernandez, Marco Pagani, Eugenio Gallina

**Affiliations:** ^1^Centro di Ricerca e Studi in Psicotraumatologia (CRSP), Milan, Italy; ^2^EMDR Italy Association, Varedo, Italy; ^3^Institute of Cognitive Sciences and Technologies, Consiglio Nazionale delle Ricerche, Rome, Italy

**Keywords:** healthcare workers, EMDR, PTSD, COVID-19, humanitarian emergency

## Abstract

**Aim:**

The Coronavirus 2019 (COVID-19) pandemic represents one of the most catastrophic events of recent times. Due to the hospitals’ emergency situation, the population of healthcare workers was the most affected. Healthcare workers who were exposed to COVID-19 patients are most likely to develop psychological distress and post-traumatic stress disorder (PTSD). The present study aimed at investigating PTSD in a sample of Italian healthcare workers during this outbreak and to evaluate the effectiveness of the Eye Movement Desensitization and Reprocessing (EMDR) Therapy with this population.

**Methods:**

A total of 744 healthcare workers were included. 587 healthcare workers were treated with EMDR, while the other 157 were not treated. Participants were asked to provide sociodemographic information; the post-traumatic symptomatology was evaluated through Impact of Event Scale-Revised (IES-R) and to investigate the level of intensity of emotional activation was used The Emotion Thermometer (THERMO) at two time points (pre-post treatment).

**Results:**

The results obtained between EMDR treatment and non-EMDR treatment were evaluated on only 2 hospitals. Treatment group *n* = 68 vs. waitlist non-treatment group *n* = 15**7.** All scores pre- and post-EMDR decreased significantly (*p* < 0.001) showing an evident effect of EMDR. The differences between pre- and post-treatment of the IES-R scores of subjects in which EMDR was performed as compared to the scores pre- and post-12 weeks of waiting list subjects in which it was not performed were significantly different (*p* < 0.001).

**Limitation:**

The emergency situation did not provide an opportunity to explore further aspects that would have been important for research. One limitation is the use and analysis of only two standardized tests. In addition, other psychopathologies were not investigated as outcome measures. A limitation is the comparison of subjects treated online and *de visu*. Although the protocol used was the same, the mode of intervention may have influenced the results. In addition, the effectiveness of EMDR treatment was only evaluated at two time points (pre-post) with no possibility of follow-up and the lack of a control group.

**Discussion/conclusion:**

The findings of the present study suggest that healthcare workers were at high risk of developing PTSD when confronted with COVID-19 outbreak and suggest the importance of psychological support during this humanitarian emergency.

## Introduction

In December 2019, a series of atypical cases of pneumonia were reported in Wuhan, China. Subsequently, the World Health Organization (WHO) defined these cases as Coronavirus 2019 (COVID-19) (Anand et al., 2020). The WHO then officially declared the outbreak as a global pandemic on February 11, 2020.

The virus has rapidly spread throughout China and elsewhere, becoming a global health emergency (World Health Organization, 2020). The rapidly evolving situation drastically altered people’s lives, with multiple consequences on the global economy, both public and private (Xiong et al., 2020). The crisis has affected different areas: tourism, transport, agriculture, industry and finance. In fact, due to the outbreak of COVID-19, governments have imposed heavy restrictions nationally and internationally (Xiong et al., 2020).

Since the first months, some phenomena related to mental health have been highlighted in healthcare workers: depressive symptoms, anxiety symptoms, associated risk factors and PTSD (Xiong et al., 2020). Taking into account studies conducted during previous viral epidemics (Chong M. Y. et al., 2004; Chong P. Y. et al., 2004; Wu et al., 2009), it is the mental health of medical and nursing staff that seems to be most at risk. So, the emergency had a negative impact on the psychological wellbeing not only of the general population but also and especially of the medical population, presenting, for the latter, multiple risk factors for the development of posttraumatic reactions (World Health Organization, 2020). These factors may have impacted medical and nursing staff in Wuhan and around the world, leading to serious and persistent mental health problems.

Specifically, in China, healthcare workers have been found to have extremely high rates of post-traumatic stress disorder (PTSD), more than 50% of healthcare workers providers in all studies exceeded the cut-off (Kang et al., 2020; Lai et al., 2020; Li et al., 2020; Liang et al., 2020; Lu et al., 2020; Mo et al., 2020; Shi et al., 2020; Wu et al., 2020).

Also in Italy, early studies found a high incidence of PTSD in 38% of healthcare workers (Di Tella et al., 2020).

Pappa et al. (2020) published data on MDs in the United Kingdom and Greece, where significant mood and sleep problems were found, demonstrating that healthcare workers employed in COVID-19 emergency are at high risk of stress, burnout and PTSD (Chirico et al., 2020).

Many studies further confirmed these findings on nurses, doctors, and general practitioners who required psychological treatments aimed at relieving stress and preventing the onset of psychological disorders (Cao et al., 2020). Given the significance of the prevalence of PTSD in the healthcare population, it is imperative to investigate the effectiveness of programs through the validation of protocols in specific emergency situations. During the COVID-19 emergency, many departments around the world ensured psychological support for healthcare workers: some interventions were based on existing disaster protocols, others created online platforms, apps and websites to help them during their difficult daily lives and others set up *defusing* sites within hospitals (Castelnuovo et al., 2020; Buselli et al., 2021).

The aim of the present research is to investigate the efficacy and the acceptability of Eye Movement Desensitization and Reprocessing (EMDR) treatment to address the immediate stress and traumatic symptoms and prevent its long-term consequences, in healthcare workers during the COVID-19 pandemic.

To assess its impact, we will compare the level of traumatization in groups of health care professionals undergoing or not EMDR. We will also compare the effects of the online-based treatment to face to face sessions of EMDR, both at individual or group level taking into account potential confounding factor as age, gender, profession etc.

The study will contribute to shed light on the correct procedures and treatments to be carried out to improve the prevention of psychological disorders in healthcare workers.

## Materials and methods

### Partners

This study was conducted by EMDR Italy Association.

The Italian EMDR Association, in fact, has provided several group and individual interventions in emergency situations with the aim of promoting wellbeing and recovery from trauma in children, adolescents and adults. In 2020, in the context of the COVID-19 emergency, the Italian EMDR Association has developed collaborations with many Italian institutions such as the Ministry of Health, the Ministry of Education and various Municipalities to carry out psychoeducation, awareness and psychology support activities focused both on the general population, HCWs, hospitals and NHS.

### Participants

Health care facilities informed all healthcare personnel of the possibility of EMDR intervention to manage the psychological distress caused by the emergency. Freely, facility health workers decided whether or not to take part in the study.

The participants are part of a convenience sample made up of 744 health professionals (medical doctors, nurses, administrative staff, intensive care support staff, psychologists). from 18 different hospitals and nursing homes involved in the COVID-19 emergency. The sample was collected throughout the pandemic emergency period starting from November 2020 until March 2021.

Participants, who were not treated with EMDR, still had a debriefing space with other psychologists. For this reason, the comparison between treated and non-treated was done only in two hospitals (ASST Rhodense), where the 157 non-treated with EMDR were compared with the 68 treated in the same hospitals. Non-treated sample cannot be defined as a control group because for ethical reasons related to the emergency situation that did not allow the absence of psychological support even for the non-EMDR-treated group.

### Intervention

The intervention protocol was designed for online and *de visu* groups of about 4–6 participants and online through EMDR-IGTP (Eye Movement Desensitization and Reprocessiong- Intergrative Group Treatment Protocol) (Jarero et al., 2006). Sessions can range from 1 to 1 h and 30 min The participants were guided through a safe/secure place exercise or breathing exercises. The EMDR-IGTP leader asked them to think about the worst part of the event (the current crisis) and then to draw that image on the paper provided. They were then asked for the related Subjective Units of Disturbance (SUD) rating and told to write the corresponding number on their picture. After that they were asked to look at their picture and to provide their own alternating bilateral stimulation with the Butterfly Hug. The participants were then instructed to draw another picture of their own choice related to the event and rate it according to its level of distress. Processing continued with the adults looking at the second picture and using the Butterfly Hug. The process was repeated twice more so that each participant drew four pictures, and provided a SUD rating for each. The final level of distress associated with the current crisis was then assessed by asking to focus on the drawing that was most disturbing and to identify the current SUD level. This number was then written on the back of the paper and was the 5th SUD rating for the session. The participants then drew a final picture that represented their future vision of themselves, along with a word or a phrase that described that picture. No SUD rating was provided for this picture. The drawing and the phrase were then paired with the Butterfly Hug. The clients were instructed to close their eyes, scan their body, and do the Butterfly Hug or grounding techniques for the stabilization. And *de visu* individual session through EMDR standard protocol (Shapiro, 2018). All type of interventions were based on EMDR therapy. Each intervention lasted 3 meetings of about 2 h each carried out over a month, about once a week.

Interventions were managed by one psychotherapist (for session) specialized in EMDR therapy with the aim to reduce the PTSD symptoms.

### Procedure

Given the mode of operation of the health care providers and the humanitarian aim of the intervention, it was not possible to implement a randomized, delayed treatment condition. Here it is necessary to focus attention on the importance of a prompt intervention vs. a rigorous and well-planned research design.

The study has a two-point pre-post design in that the questionnaires were administered before (T0) and at the end of the intervention (T1). The first assessment took place for all participants at the beginning of the first meeting while the second at the end of the intervention.

Clinicians were responsible for pre-post assessments but data were collected and analyzed anonymously by other researchers who were doing the data analysis, in this way outcome assessor was masked.

Each participant has read and signed the informed consent and the privacy policy. Once treatment was allowed, subjects had the freedom to leave the study and psychological support at any time. Data were collected anonymously.

### Tools

The assessment protocol was based on 2 self-report questionnaires.

The characteristics of the sample were studied through *ad hoc* questions: (1) socio-demographic (age, sex, number of children, number of cohabitants); (2) job-related information (e.g., workplace and occupation). The post-traumatic symptomatology was evaluated through Impact of Event Scale-Revised (IES-R) in accordance with the criteria of the DSM IV-TR (Weiss and Marmar, 1997) validated and translated into Italian (Pietrantonio et al., 2003). The latest PCL-5 (American Psychiatric Association, 2013) was not used, as it is free (in Italian version) as of May 2019. We considered it late and for the reasons of homogeneity we always did the IES-R. This psychometric test consists of 22 items. It includes 3 subscales measuring the following dimensions: intrusion, avoidance and hyperactivation. Participants were asked to rate their level of post-traumatic symptoms using a 5-point Likert scale ranging from 0 (= “not at all”) to 4 (= “a lot”) referring to the previous 7 days. The total score between 0 and 88. The cut-off of 33 highlights a high risk of PTSD; in line with the literature, there are no specific cut-offs for scale interpretations. The Italian translation of IES-R has shown satisfactory internal validity in studies on different populations at risk, as reported by Craparo et al. (2013) (Intrusion, α = 0.78; Avoidance, α = 0.72; Hyperarousal, α = 0.83) and Converso and Viotti (2014) (Intrusion, α = 0.91; Avoidance, α = 0, 81; Hyperarousal, α = 0.87). Although the IES-R has not been validated in the general Italian population, it has been used to evaluate the symptomatology of PTSD in many Italian samples, which confirmed its adequate reliability (Gambetti et al., 2011; Priebe et al., 2011; Maslovaric et al., 2017).

The Emotion Thermometer (THERMO, Mitchell et al., 2010), a visual analog self-assessment scale to collect the level of intensity of emotional activation on a Likert scale from 1 to 10 regarding some main emotional experiences (stress, depressed mood, anxiety, anger, sleep problems, need for help) during the previous week was also submitted to the investigated subjects.

### Statistical analysis

All the original variables were described in terms of their basic location and variability indexes. The binary variables (sex, treatment) were coded as: 0 = female, 1 = male; and 0 = non-treated, 1 = EMDR treated, respectively, implying that the mean of binary variables corresponds to the relative proportion of males and EMDR treated, respectively. The same descriptive indexes were applied to derived variables, namely the “delta” variables, corresponding to the difference between pre and post treatment scores for IES and THERMO.

Inferential statistics on the delta variables testing the null hypothesis of delta = 0, equivalent to a paired test paradigm, was computed by means of two non-parametric (Sign and Signed-Rank) and one parametric (*t*-test) approach. The very high number of subjects allows to consider both parametric and non-parametric procedures as apt to the evaluation of statistical significance (Edgell and Noon, 1984), on the other hand, the high number of subjects generates a very high statistical power with a consequent possible burden of statistically significant results correspondent to clinically not-relevant differences (Kraemer et al., 2003).

In the case of the two centers in which both treated and non-treated groups were present, the statistical significance between the two groups as for delta variables (entity of the pre-post differences) was estimated by a non-parametric approach based on Wilcoxon scores evaluated by a chi-square Kruskal-Wallis test (Iman and Davenport, 1976).

Principal Component Analysis (PCA) as applied to delta values for both IES and THERMO variables generated two synthetic indexes (PC1DELTAIES and PC1DELTATHERMO) explaining the coherent part of the two test paradigms and thus allowing for a global estimation of treatment effect (Giuliani, 2017).

The between variables correlation was estimated by means of Spearman correlation coefficient, while the modulatory effects of sex, living condition (domicilio), Treatment Center (Ospedale) and Kind of Therapy were estimated by means of an Analysis of Variance (ANOVA) approach.

## Results

Despite the large unbalance between females and males there were no difference in response to EMDR between the two genders.

Of the total sample of 744 health professionals 587 were treated with EMDR (433 females and 154 males, mean age 45.5 ± 9.9 years) and 157 were not treated (125 females and 27 males, mean age 44.8 ± 10.6 years).

Overall, 744 subjects completed the IES-R before undergoing EMDR and 706 post-EMDR. The differential attrition in this study was less than 15%.

The results of the scores of the three constructs and the total scores of the test are reported in Table 1.

**TABLE 1:** EMDR treatment.

*N* = 706	(M/SD) pre	(M/SD) post	Delta *p* <
Tot IES-R	39.13/17.62	21.63/17.605	0.001
Avoidance	12.41/6.318	7.89/6.623	0.001
Intrusiveness	15.35/7.272	8.06/6.879	0.001
Hyperarousal	11.34/5.985	5.74/5.361	0.001

All Deltas between the scores pre- and post-EMDR decreased significantly (*p* < 0.001) showing an evident effect of EMDR. It is worth noting that 68% of the investigated subjects had a pathological pre EMDR IES-R score (>33).

Likewise, 442 subjects completed the Emotions Thermometer pre-EMDR and 338 post-EMDR with highly significant Deltas between the scores of all dimensions (Table 2).

**TABLE 2:** THERMO.

*N* = 442	Stress	Anxiety	Mood	Angry	Sleep	Help
**PRE**						
Mean	5.27	4.42	3.51	4.04	4.36	3.79
SD	2.594	2.825	2.780	3.103	3.205	2.695
*N* = 338						
**POST**						
Mean	3.22	2.47	1.95	2.43	2.49	2.33
SD	2.265	2.126	2.079	2.323	2.689	2.247
*Delta p* <	0.001	0.001	0.001	0.001	0.001	0.001

Summing up, all variables showed a significant improvement after EMDR demonstrating a clear effect of treatment.

In order to properly compare treated and non-treated subjects, we analyzed only the data from the two structures in which both populations were present, with the statistical benefit of keeping under control all possible confounding variables. The Deltas between pre- and post-treatment of the IES-R scores and THERMO of subjects in which EMDR was performed (treated, *n* = 68) as compared to the scores pre- and post-12 weeks of waiting list subjects in which it was not performed (non-treated, *n* = 157) was significantly different (Tables 3, 4), speaking in favor of a much stronger effect of treatment vs. mere passage of time.

**TABLE 3:** IES.

	Avoidance	Intrusiveness	Hyperarousal	Tot
Δ Not treat				
*N* = 157				
Mean	5.1	6.4	4.9	16.4
SD	8.3	8.2	6.4	21.5
Δ Treat				
*N* = 68				
Mean	8.9	12.1	8.9	29.8
SD	7.0	7.9	5.7	18.5
*Difference delta P* <	0.001	0.001	0.001	0.001

**TABLE 4:** THERMO.

	Stress	Anxiety	Mood	Angry	Sleep	Help
Δ Not treat						
*N* = 157						
Mean	1.7	1.7	1.5	1.5	1.7	1.2
SD	2.6	2.7	2.3	3.2	2.7	2.2
Δ Treat						
*N* = 68						
Mean	2.8	2.5	2.8	2.3	2.8	2.0
SD	2.4	2.2	2.3	2.3	2.8	2.8
Difference delta *P* <	0.005	NS	0.001	NS	NS	0.05

Furthermore, 31 out of 157 non-treated subjects (about 20% of the total) showed a worsening after 12 weeks but only 2 of the 68 treated (about 3%) did it.

## Discussion

The present study aimed to investigate the efficacy and the acceptability of EMDR treatment to address the immediate stress and traumatic symptoms and prevent its long-term consequences, in healthcare workers during the COVID-19 pandemic.

Regarding the efficacy of EMDR intervention, the present study showed that there was a significant difference in IES-R and THERMO scores between pre- and post-intervention. In fact, all Deltas between the scores pre- and post-EMDR decreased significantly (*p* < 0.001) showing an evident effect of EMDR. It is worth noting that 68% of the investigated subjects had a pathological pre EMDR IES-R score (>33).

Our results are supported by the scores of the study by Lai et al. (2020) in which more than 50% of the sample had scores above the threshold at the IES-R. The study showed that 71.5% among physicians and nurses had symptoms of traumatic stress and the level was moderate/severe in 35% of them. Specifically, 33% of physicians and 36.2% of nurses had clinically relevant symptoms (Lai et al., 2020).

The assessment was specifically assessed through the Impact of Event Scale-Revised (IES-R) instrument.

This tool was also used by other studies that investigated the psychological impact of COVID-19 related trauma in healthcare workers (Chew et al., 2020; Kang et al., 2020; Lai et al., 2020; Tan et al., 2020; Zhang et al., 2020). These studies showed the following percentages of PTSD: 7.5% in the study by Chew et al. (2020), 60% in Kang et al. (2020), 7.7% in Tan et al. (2020), and 73.4% in Zhang et al. (2020). The differences in percentages can be attributed to sample differences as explained below. In contrast to this research which adhered to the traditional scoring of presence or non-presence of post-traumatic symptoms, these studies, done in China, interpreted the IES-R scores as follows: normal/sub-clinical (0–8), mild (9–25), moderate (26–43) and severe distress (44–88), with cut-off of 26 (Kang et al., 2020; Lai et al., 2020; Zhang et al., 2020). While studies conducted in Singapore and India assessed IES-R scores as follows: normal (0–23), mild (23–32), moderate (33–36) and severe (>37), with cut-off of 24 indicating possible PTSD (Chew et al., 2020; Tan et al., 2020).

HCWs had therefore found themselves facing critical situations that increase the risk of suffering psychologically, deriving from facing various dangerous conditions, with consequences that could extend from psychological distress to mental health symptoms like stress, depressed mood, anxiety, anger, sleep problems and need for help. These areas were evaluated with THERMO and all Deltas between the scores pre- and post-EMDR decreased significantly (*p* < 0.001) showing an evident effect of EMDR for these symptoms.

According to literature In China, HCWs have been found to have extremely high rates of depression, generalized anxiety disorder (GAD), insomnia, stress-related symptoms and PTSD (Cao et al., 2020; Kang et al., 2020; Lai et al., 2020; Li et al., 2020; Liang et al., 2020; Lu et al., 2020; Mo et al., 2020; Shi et al., 2020; Wu et al., 2020).

In Germany, doctors (MDs) have found high levels of anxious and depressive symptoms (Bohlken et al., 2020).

As part of this pandemic, Pappa et al. (2020) published data on MDs in the United Kingdom and Greece, where significant mood and sleep problems were found, demonstrating that healthcare workers employed in the COVID-19 emergency are at high risk of stress, burn-out and disturbance from post-traumatic stress (Chirico et al., 2020).

Also in Italy, the first studies found important psychological illnesses such as depressive symptoms and post-traumatic stress symptoms on health workers (Di Tella et al., 2020).

Then, in order to properly compare treated and non-treated subjects, the Deltas between pre- and post-treatment of the IES-R and THERMO scores of subjects in which EMDR was performed as compared to the scores pre- and post-12 weeks of waiting list subjects in which it was not performed was significantly different (*p* < 0.001), speaking in favor of a much stronger effect of treatment vs. mere passage of time.

As far as the literature is concerned, the intervention on healthcare personnel exposed to the COVID-19 pandemic has not yet been investigated in depth, although studies have already been collected on the protocols implemented during this global crisis (Buselli et al., 2021). Specific interventions on healthcare workers with EMDR treatment during COVID-19 are still absent.

The results of the present study could contribute to shed light on the correct procedures and treatments to be carried out to improve the prevention of psychological disorders in healthcare workers and demonstrate how healthcare workers and how psychological support, through EMDR treatment, improve the prevention of psychological disorders is effective (Shapiro, 2001; Maslovaric and Fernandez, 2016). EMDR therapy is a brief intervention and in this study we have observed how in only three group meetings of 2 h each the level of symptomatology decreased significantly. This is in line with the study of Mavranezouli et al. (2020), where EMDR was found to be the most cost-effective treatment for PTSD (less sessions and high level of effectiveness).

In conclusion, this study allows both to hypothesize the effectiveness of EMDR intervention on healthcare workers but also to hypothesize that other psychological support helps in the reduction of traumatic symptomatology and symptoms such as stress, depressed mood, anxiety, anger, sleep problems, need for help. This is a helpful intervention since these health care workers will continue to be exposed to triggers that can reactivate and remind the most traumatic images they experienced during the Pandemic. To have an effective intervention in the acute phase can give immediate relief, prevent chronization and enhance resources and resilience for future situations. The high level of traumatization in the personnel after the Pandemic found in this study would have been a strong risk factor in general for the mental health and functioning in the workplace. To offer an intervention such as EMDR in the acute phase can also prevent costs in terms of the organization, personnel and at a subjective and family level (Shapiro, 2012).

However, especially in the field of emergencies, which are characterized by a series of challenges due to the implicit characteristics of the event, such as unpredictability and ethical implications that force a sudden intervention, there is an important difficulty in monitoring the outcomes of the intervention and scientific research. For this reason, in the following section, the limitations present in the present research have been exposed.

## Potentials and limitations

This study has the potentiality of having been carried out in a moment of difficulty due to the Italian situation during the pandemic. In fact, the opportunity to have drawn up a pre-post study during a period of crisis allows us to observe a partial psychological situation of a given population, in a given historical period. The emergency situation did not give the possibility to deepen further aspects that would have been important for the research, however it was possible to have a not treatment group, in order to understand the effectiveness of the EMDR protocol on a specific population.

Although the results of the present study are encouraging several limitations are present.

A limitation is represented by the use and analysis of only two standardized tests. In addition, other psychopathologies were not investigated as an outcome measure. The administration of other psychometric tests for the assessment of other psychopathologies may be functional for future research.

Although functional and dysfunctional coping strategies adopted by practitioners during the Pandemic have not been evaluated, this could be a good starting point for a future study.

One bias is definitely the gender distribution in the sample, with a large female prevalence.

One limitation is the comparison of subjects treated online and *de visu*. Although the protocol used is the same, the mode of intervention may have affected the outcomes.

Moreover, the efficacy of EMDR treatment was evaluated in only two times (pre-post) without a possibility of *follow up* and, therefore, the absence of a longitudinal control aimed at following the reduction of PTSD symptoms over time.

A further limitation is related to the no treat group, as it was selected from only two hospitals.

Certainly, these limitations reduce the generalization of the results and may have affected the study.

## Conclusion

COVID-19 had a significant impact on the wellbeing of healthcare workers that need for mental health protection, support, and treatment. This study demonstrated that interventions with EMDR for this population had a positive effect to significantly decrease symptoms such as stress, depressed mood, anxiety, anger, sleep problems and need for help.

This confirms that working with EMDR in emergency situations provokes immediate relief, prevents chronization.

Also, the study confirmed that EMDR protocol in all its modalities, both online and *de visu*, can protect health care workers from the consequences of acute stress.

In conclusion, the possibility in the future of collecting further data may improve the statistical strength of the study and observe the resilience of a specific population as time goes on, in order to understand if an early intervention with EMDR, during a critical event, can help the growth of this evolutionary skill.

## Data availability statement

The raw data supporting the conclusions of this article will be made available by the authors, without undue reservation.

## Ethics statement

Ethical review and approval was not required for the study on human participants in accordance with the local legislation and institutional requirements. The patients/participants provided their written informed consent to participate in this study.

## Author contributions

IF, EG, and MP contributed to conception and design of the study and wrote the first draft of the manuscript. EG organized the database. MP performed the statistical analysis. All authors wrote sections of the manuscript, contributed to manuscript revision, read, and approved the submitted version.
